# Short‐term use of oral amiodarone causing torsades de pointes

**DOI:** 10.1002/ccr3.1659

**Published:** 2018-06-22

**Authors:** Stephen Yau, Patrick Chan, Joshua Sapkin, Eric Hsieh

**Affiliations:** ^1^ Department of Internal Medicine University of Southern California Los Angeles CA USA

**Keywords:** amiodarone, antiarrhythmic, polymorphic, tdp, torsades de pointes, ventricular tachycardia

## Abstract

Amiodarone is one of the most commonly used antiarrhythmic drugs. Despite its well‐known side effects, amiodarone is considered to be a relatively safe drug, especially in short‐term usage to prevent life‐threatening ventricular arrhythmias. Our case demonstrates an instance where short‐term usage can yield drug side effect.

## 1 INTRODUCTION

Amiodarone is one of the most commonly used antiarrhythmic drugs. Despite its myriad well‐known side effects, which are often associated with long‐term usage, amiodarone is considered to be a relatively safe drug, especially in short‐term usage that can prevent life‐threatening ventricular arrhythmias. The potential for short term, potentially fatal consequences is often forgotten.

This case describes a relatively healthy, young patient who was admitted to the cardiac intensive care unit (CCU) with suspected monomorphic VT arrest who was initially incorrectly treated as such, with subsequent change to the correct treatment with resolution of symptoms.

## 2 CASE

A 44‐year‐old man with a history of HTN, DM type 2, and recent diagnosis of atrial fibrillation was seen in clinic and initiated on amiodarone with planned direct current (DC) cardioversion in 4 weeks. Ten days after beginning amiodarone, he presented to the emergency room after four syncopal events while sitting on his couch at home. All episodes occurred with spontaneous recovery. EKG on admission revealed atrial fibrillation with a heart rate in the 50s and a QTc of 500 ms. Furthermore, magnesium (Mg) upon admission was 1.4.

**Figure 1:**
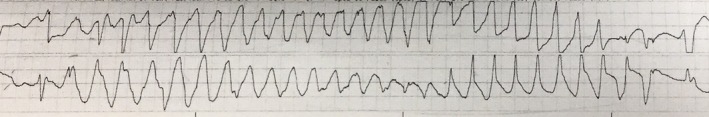
Captured rhythm strip after initiation of amiodarone bolus and drip, depicting polymorphic ventricular tachycardia, that is, torsades de pointes

Shortly after admission, he had a witnessed episode of ventricular tachycardia (VT) arrest and required defibrillation prior to achieving return of spontaneous circulation (ROSC). He was subsequently administered an amiodarone bolus and started on an amiodarone drip. A few hours later after admission to the CCU, he had an episode of VT arrest that resolved without any intervention. At this time, the printed rhythm strip was reviewed and significant for polymorphic ventricular tachycardia, aka Torsades de Pointes (TdP) (Figure 1). The correct treatment was initiated including immediate discontinuation of amiodarone, aggressive magnesium repletion, followed by isoproterenol intravenously to increase the HR to >70. Telemetry monitoring for the next 5 days did not demonstrate further evidence of sustained VT and the patient remained asymptomatic. An echo was performed during admission with EF 50%, normal left ventricular cavity size, no regional wall motion abnormalities, a suboptimal EKG exercise stress test was performed that revealed no PVCs and a subsequent pharmacologic nuclear stress test was also performed that admission without evidence of ischemia. Moreover, the patient was discharged. At clinic follow‐up 1 month later, the patient remained asymptomatic without further syncopal episodes.

## 3 DISCUSSION

This case reiterates the importance of remembering that antiarrhythmics, particularly Class III (potassium channel blockers),^1^ are also proarrhythmic with the possibility for immediate fatal consequences. By delaying repolarization and increasing the action potential duration, the QTc interval is increased and there is an elevated risk of developing TdP via early after depolarizations (EAD). Although the incidence of amiodarone‐induced TdP is <1%^2^ and is more common in other class III drugs, such as sotalol and ibutilide, this case further illustrates the point that antiarrhythmic drugs are also proarrhythmic. Risk factors include female sex (most common), bradycardia, underlying heart disease, prolonged QTc at baseline, and electrolyte disturbances (hypokalemia, hypomagnesemia).^3^

**Figure 2:**
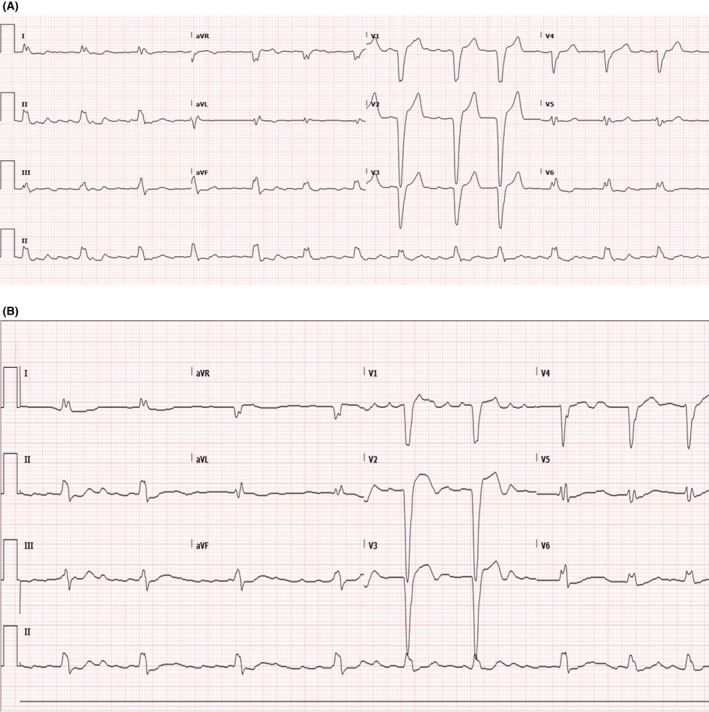
EKGs from before and after initiation of oral amiodarone. A, Before with a HR of 80, left bundle branch block, atrial fibrillation and an intrinsic prolonged QTc of ~490. B, After with profound bradycardia with a HR of 52, left bundle branch block, atrial fibrillation, and QTc of ~500

In this case, the patient may have already had an intrinsically prolonged QTc (albeit, in the setting of atrial fibrillation and an intrinsic left bundle branch block makes accurate calculation much more difficult),^3^ worsened with amiodarone, a subsequent bradycardia (Figure 2) after initiation of amiodarone^4^ (amiodarone acts via sodium, potassium, and calcium channel blockade and also causes AV nodal blockade which can lead to profound bradycardia), and a decreased Mg on admission. Together, this constellation of problems contributed to this patient's rare presentation of amiodarone‐induced TdP.

This case also demonstrates the importance of looking at a patient's complete picture rather than reflexively resorting to an antiarrhythmic drug for ventricular arrhythmias. There were many signs and symptoms that could have prevented further amiodarone toxicity on initial arrival to the ED, including recurrent syncope that resolved spontaneously in the setting of new onset bradycardia and worsening QTc prolongation shortly after starting oral amiodarone.

## CONFLICT OF INTEREST

None declared.

## AUTHORSHIP

SY: Wrote original manuscript, researched topic and collected data, on service and took care of patient during admission. PC: Wrote original manuscript, researched topic and collected data. JS: Helped with research, edited manuscript. EH: Edited manuscript, supervised and coordinated case report.
